# Determination of MLC model parameters for Monaco using commercial diode arrays

**DOI:** 10.1120/jacmp.v17i4.6190

**Published:** 2016-07-08

**Authors:** Paul Kinsella, Laura Shields, Patrick McCavana, Brendan McClean, Brian Langan

**Affiliations:** ^1^ Physics Department Saint Luke's Hospital Dublin Ireland; ^2^ School of Physics, Science Centre ‐ North, University College Dublin Belfield Dublin Ireland

**Keywords:** Monaco, MapCHECK 2, ArcCHECK, transmission probability filter, MLC

## Abstract

Multileaf collimators (MLCs) need to be characterized accurately in treatment planning systems to facilitate accurate intensity‐modulated radiation therapy (IMRT) and volumetric‐modulated arc therapy (VMAT). The aim of this study was to examine the use of MapCHECK 2 and ArcCHECK diode arrays for optimizing MLC parameters in Monaco X‐ray voxel Monte Carlo (XVMC) dose calculation algorithm. A series of radiation test beams designed to evaluate MLC model parameters were delivered to MapCHECK 2, ArcCHECK, and EBT3 Gafchromic film for comparison. Initial comparison of the calculated and ArcCHECK‐measured dose distributions revealed it was unclear how to change the MLC parameters to gain agreement. This ambiguity arose due to an insufficient sampling of the test field dose distributions and unexpected discrepancies in the open parts of some test fields. Consequently, the XVMC MLC parameters were optimized based on MapCHECK 2 measurements. Gafchromic EBT3 film was used to verify the accuracy of MapCHECK 2 measured dose distributions. It was found that adjustment of the MLC parameters from their default values resulted in improved global gamma analysis pass rates for MapCHECK 2 measurements versus calculated dose. The lowest pass rate of any MLC‐modulated test beam improved from 68.5% to 93.5% with 3% and 2 mm gamma criteria. Given the close agreement of the optimized model to both MapCHECK 2 and film, the optimized model was used as a benchmark to highlight the relatively large discrepancies in some of the test field dose distributions found with ArcCHECK. Comparison between the optimized model‐calculated dose and ArcCHECK‐measured dose resulted in global gamma pass rates which ranged from 70.0%–97.9% for gamma criteria of 3% and 2 mm. The simple square fields yielded high pass rates. The lower gamma pass rates were attributed to the ArcCHECK overestimating the dose in‐field for the rectangular test fields whose long axis was parallel to the long axis of the ArcCHECK. Considering ArcCHECK measurement issues and the lower gamma pass rates for the MLC‐modulated test beams, it was concluded that MapCHECK 2 was a more suitable detector than ArcCHECK for the optimization process.

PACS number(s): 87.55.Qr

## I. INTRODUCTION

The modeling of multileaf collimator (MLC) transmission is more important for intensity‐modulated radiation therapy (IMRT) and volumetric‐modulated arc therapy (VMAT) than for 3D conformal radiation therapy (3D CRT) as the MLCs shadow the treatment area for a large

fraction of the delivered monitor units (MU).^1^, ^2^ Consequently, MLCs need to be characterized accurately in treatment planning systems (TPSs) to facilitate accurate IMRT and VMAT. Nelms et al.^(3)^ showed how patient‐specific quality assurance (PSQA) procedures fail to highlight errors in the MLC model, indicating the necessity to characterize them correctly at the time of commissioning.

Monaco TPS (IMPAC Medical Systems, Inc., Sunnyvale, CA (an affiliate of Elekta AB, Stockholm, Sweden)) employs the X‐ray voxel Monte Carlo (XVMC) dose calculation algorithm. The jaws and MLCs are characterized in the XVMC model using a transmission probability filter (TPF).^4^, ^5^, ^6^, ^7^ While commissioning and dosimetric evaluation of Monaco XVMC has been previously described,^5^, ^6^, ^8^, ^9^, ^10^, ^11^ no specific details were provided on how the TPF MLC characteristics were determined. Monaco TPF possesses a number of MLC parameters that can be adjusted based on measurements of test fields with the users PSQA measurement device as recommended by the vendor.^(12)^ AAPM TG‐106^(13)^ has suggested the use of film, portal images or diodes as measurement devices to determine MLC characteristics. The aim of this study was to explore if it was possible to optimize the TPF MLC parameters of the XVMC dose calculation algorithm and to evaluate the relative merit of MapCHECK 2 and ArcCHECK for this process.

## II. MATERIALS AND METHODS

**Figure 1:**
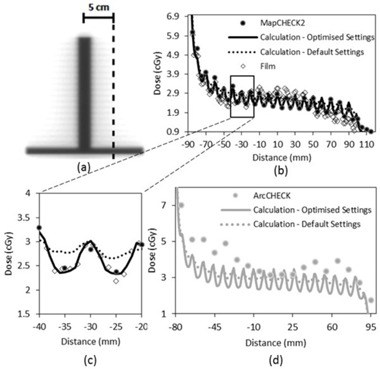
Dose distributions resulting from the T‐shaped test beam: (a) planar greyscale dose distribution; (b) and (d) dose profiles along the dashed line in (a). The profile in (c) corresponds to the highlighted rectangular region of interest in (b). The data labels in (b). 1a.lsDoo apply in (c).

A 6 MV XVMC model of an Elekta Synergy linac (Elekta Limited, Crawley, UK) equipped with an MLCi treatment head was established in Monaco Version 5.00. The description of the XVMC beam model can be divided into patient‐independent and patient‐dependent components.^(6)^ The patient‐independent component describes the radiation from three separately modeled virtual sources in the treatment head: primary photon, secondary photon, and electron contamination. The jaws and MLCs are patient plan‐dependent and are characterized using a TPF which facilitates faster calculation times.^(4)^ The TPF is characterized by geometric and probabilistic parameters, where the geometric parameters describe the dimensions of the collimators while the probabilistic parameters describe the probability of transmission through different parts of the collimators. Table 1 lists the adjustable TPF MLC parameters in Monaco. A collection of test fields supplied with the TPS (Table 2) were used to evaluate the XVMC MLC model. The AAPM IMRT subcommittee recommends that when evaluating MLC transmission a large region should be sampled.^(1)^ The FOURL test beam described in Table 2 sampled an area of transmission of approximately $4\times 4\;{\text{cm}}^{2}$. This relatively small region was not found to adequately sample MLC transmission, so a T‐shape field was created instead using the MLCs to form two large transmission regions (Fig. 1(a)).

Film and diodes have been recommended by Report 22 of the Netherlands Commission on Radiation Dosimetry^(14)^ and AAPM TG‐106^(13)^ for evaluating finer detail of MLC characteristics, for example discrimination between interleaf and intraleaf leakage. It was recommended by the manufacturer to measure the test fields described in Table 2 with a PSQA measurement device to enable optimization of the TPF MLC model parameters. The T‐shape field and test fields from Table 2 were measured with two PSQA diode arrays: MapCHECK 2 and ArcCHECK (Sun Nuclear Corp., Melbourne, FL). The leaf transmission, interleaf leakage, leaf offset, MLC corner leakage, and leaf groove width were iteratively adjusted to improve the match between MapCHECK 2 measurements and Monaco calculations for a selection of the test beams. MapCHECK 2 was chosen as the reference detector because initial comparison of the test field calculated and ArcCHECK‐measured dose distributions revealed that it was unclear how to change the MLC parameters to gain agreement. The ambiguity arose with ArcCHECK due to an insufficient sampling resolution and unexpected discrepancies in the dose distributions which will be highlighted in the Results and Discussion sections. The test fields were subsequently delivered to Gafchromic EBT3 film (Ashland Specialty Products, Wayne, NJ) to independently verify MapCHECK 2 measurements by visual inspection. Dose calculations with the default MLC parameters are referred to as the ‘Default model’ and calculations with the adjusted MLC parameters are referred to as the ‘Optimized model’.

**Table 1:** TPF MLC parameters in Monaco 5.0

*TPF‐MLC Parameter*	*Default Setting*	*Optimized Setting*
Leaf transmission	0.0120	0.007
Leaf groove width (mm)	1.0	1.2
Interleaf leakage	5.00	25
Leaf tip Leakage	1.10	1.10
Leaf offset (mm)	0	−0.10
MLC corner leakage	0	0.1

**Table 2:** Manufacturer‐supplied test beams for MLC model evaluation

*Beam Name*	*Description & Purpose*	
10×10	$10\times 10\;{\text{cm}}^{2}$ open field. The purpose of this field was to assess the calibration of the measurement device.	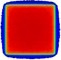
20×20	$20\times 20\;{\text{cm}}^{2}$ open field. The purpose of this field was to assess the response of the QA device to a large field and to examine for the presence of beam asymmetry.	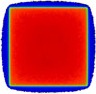
3ABUT	Three 6 cm wide fields via step‐and‐shoot delivery. The fields were designed to abut and created two junctions. This plan was used to evaluate MLC calibration and the leaf offset parameter.	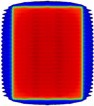
7SEGA	Seven 2 cm wide fields via step‐and‐shoot delivery. The fields were designed to abut and created six junctions. This plan was used to evaluate MLC calibration and the leaf offset parameter.	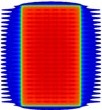
FOURL	Four L‐shaped fields via step‐and‐shoot delivery. Each sequential L is smaller producing an overall large L‐shape distribution. The plan was designed to ‘abut’ on each side of the L‐shape. This field was used to examine the leaf position offset, the MLC transmission, and the MLC groove width settings.	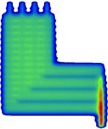
DMLC	A dynamic 10 cm sweep with a 2 cm wide field (defined by both the leaves and backup jaws). The dose distribution was sensitive to changes in the MLC position offset, transmission, and calibration.	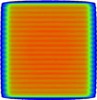
HIMRT	Head and neck step‐and‐shoot IMRT field. The intention of this field was to evaluate the impact the TPF MLC settings in a complex clinical situation.	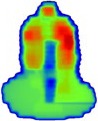
HDMLC	Head and neck sliding window IMRT field. The intention of this field was to evaluate the impact the TPF MLC settings in a complex clinical situation.	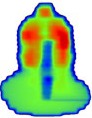

Film could not be used to verify ArcCHECK measurements in the same way it was used for MapCHECK 2 because it was not possible to take measurements with film in the same conditions as the detectors in ArcCHECK because a phantom that mimics ArcCHECK was unavailable. Consequently, the test beam dose distributions calculated with the optimized and default models were compared with ArcCHECK measurements to highlight the differences found. The diode array measured doses were compared to the calculated dose via 2D gamma analysis with SNC patient software version 6 (Sun Nuclear Corp.) to quantitatively demonstrate the improvements in the model after adjustment of the MLC parameters and the discrepancies found with ArcCHECK. Unless stated otherwise, global gamma analyses with a 10% dose threshold were performed throughout this study.

All MapCHECK 2 measurements were set up with a source‐to‐detector distance (SDD) of 100.0 cm with 8 cm of Solid Water Model 457 (Gammex, WI) placed on top. All MapCHECK 2 measurements were taken with the detector array centered, then shifted 0.5 cm (perpendicular to the MLC motion) and combined using the merge function in SNC patient software, thereby increasing the sampling resolution. All ArcCHECK measurements were acquired with the device centered on the radiation isocenter using the in‐room lasers and with the ArcCHECK cavity plug and solid insert *in situ*. The ArcCHECK ‘Merge (for higher density)’ feature was used for all ArcCHECK measurements to improve the diagonal sampling resolution of the data from 1.4 to 0.7 cm.^(15)^ This method involved taking measurements with the ArcCHECK rotated 2.72° and shifted along the long axis of the device by 5 mm. Permanent marks on the device surface and an in‐built inclinometer facilitated precise setup. The rotated ArcCHECK measurements were then ‘merged’ with the unrotated measurements in the SNC patient software to produce a higher density of measurement points.

Gafchromic EBT3 film was calibrated against a Farmer‐type ionization chamber using the triple channel dosimetry method.^(16)^ The test fields (Table 2) were delivered to the EBT3 film which was positioned at 90 cm SSD with 10 cm buildup and 5 cm backscatter of Solid Water Model 457. The film was scanned using the single scan protocol^(16)^ on an Epson Expression 10000 XL scanner (US Epson, Long Beach, CA) using the recommended scanning resolution of 72 dpi in a 48‐bit RGB format.^16^, ^17^, ^18^ Glass was placed over the calibration and test film during scanning to minimize Newton's rings artifacts. Lateral displacement of the film within the scanner can cause deterioration in the accuracy of the dose measured with greater deterioration the further the film is from the center of the scanner.^(17)^ Deviations resulting from the lateral displacement were observed on film dose profiles from the ‘20×20’ test beam. Consequently, the regions of interest under consideration on each irradiated film were placed along the center of the scanner bed to minimize the lateral displacement artifact. Relevant dose profiles were extracted using FilmQA Pro (Ashland Inc., Covington, KY). The film dose profiles were plotted together with MapCHECK 2 and the calculated dose profiles and visually inspected for discrepancies.

The test beams were calculated with the XVMC model on an artificial water phantom of $50\times 50\times 50\;{\text{cm}}^{3}$ using the same geometric setup as the film and MapCHECK 2 measurements.

The relative electron density (RED) of the phantom was set to 1.000. ArcCHECK was defined in Monaco as a uniform density cylindrical phantom using a DICOM CT dataset supplied by Sun Nuclear. The RED of the ArcCHECK phantom was determined following manufacturer guidelines by iteratively adjusting the RED until the ratio of calculated doses at the entrance and exit side of the ArcCHECK phantom matched the ArcCHECK‐measured entrance‐to‐exit ratio for a 10×10 field. The calculated and measured ratios matched to less than 0.5% when the RED was set to 1.153. The dose grid size and statistical uncertainty were set to 0.15 cm and 0.5%, respectively, for all dose calculations.

## III. RESULTS

### A. Leaf transmission & intraleaf leakage adjustment using the T‐shape field

Figure 1(b) illustrates the overall agreement between MapCHECK 2, film measurements, and calculated dose in the transmission region of the T‐shape field. A representative cut‐out of Fig. 1(b) is given in Fig. 1(c) and highlights that MapCHECK 2 measurements sampled the peaks and troughs of the MLC leakage at regular intervals, which facilitated straightforward evaluation of how much the TPF MLC parameters needed to be adjusted. Both film and MapCHECK 2 measurements indicated there was a greater difference between the interleaf and intraleaf transmission compared to the calculated dose with the default settings. It was found through an iterative process that the best match between calculated and measured dose distributions resulted when the ‘leaf transmission’ parameter was adjusted from the default value of 0.0120 to 0.007 and the ‘interleaf leakage’ parameter was adjusted from default value of 5.00 to 25.00. The improvements between the calculated dose using the optimized model and the measured dose in the ‘troughs’ can be seen in Fig. 1(c). Table 3 shows that the number of points passing the gamma analysis on the T‐shape field increased from 55.2% to 82.2% for MapCHECK 2 measurements with 2% and 2 mm gamma criteria (2% dose threshold) after adjustment of the leaf transmission and intraleaf leakage parameters. Figure 1(d) shows that the agreement between the optimized model and ArcCHECK was poor, consistent with a gamma pass rate of 31.4% at 2% and 2 mm gamma criteria (2% dose threshold) (Table 3). ArcCHECK measurements were, on average, higher than the optimized model and sampled the MLC leakage peaks and troughs irregularly.

**Table 3:** Gamma analysis results for measured vs. calculated test beam doses

	*MapCHECK 2*				*ArcCHECK*			
*γ‐Criteria*	2%/2 mm		3%/2 mm		2%/2 mm		3%/2 mm	
*Field Name*	*Default*	*Optimized*	*Default*	*Optimized*	*Default*	*Optimized*	*Default*	*Optimized*
10×10	100.0	100.0	100.0	100.0	99.0	98.8	99.2	99.0
20×20	97.0	99.8	100.0	100.0	97.7	98.3	99.1	99.1
T‐shape	55.2^a^	82.2^a^	60.9^a^	84.8^a^	35.6^a^	31.4^a^	38.7^a^	33.4^a^
3ABUT	89.8	98.7	97.2	99.9	70.0	58.3	86.9	80.8
7SEGA	63.4	93.4	86.8	97.8	62.0	43.5	80.2	70.0
DMLC	64.5	80.3	68.5	97.1	68.5	66.8	85.8	84.7
HIMRT	98.7	99.6	99.6	100.0	94.7	93.4	97.8	97.0
HDMLC	98.6	99.4	99.3	99.9	95.5	95.1	98.2	97.9
FOURL	86.8	91.8	89.4	93.5	84.4	87.7	92.6	95.2

a Local gamma analysis with 2% dose threshold.

### B. Leaf offset and MLC corner leakage adjustment using the 3ABUT test field

**Figure 2:**
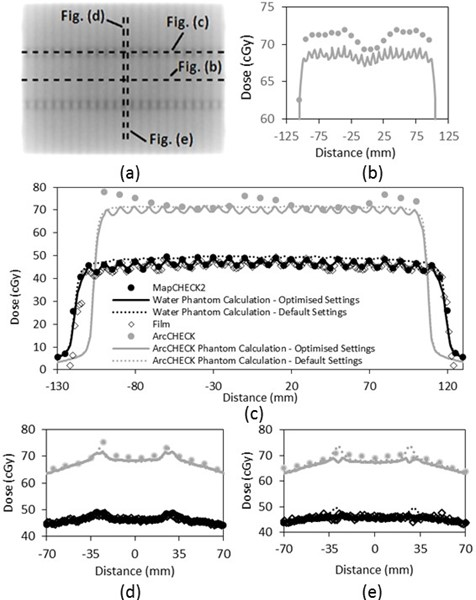
Dose distributions resulting from the 3ABUT test beam: (a) planar greyscale dose distribution; (b)–(e): dose profiles along the dashed lines in (a). The data labels in (c) apply to (b), (d), and (e).

The dose at the junctions formed by the leaf tips in the 3ABUT, 7SEGA, and FOURL test beams was affected by two adjustable TPF MLC parameters: leaf offset and MLC corner leakage. The best match between film‐measured dose and calculated dose at the junctions was found by adjusting the leaf offset from 0.0 to −0.1 mm and the MLC corner leakage from 0 to 0.01. The 3ABUT test beam was found to provide greatest clarity for adjustment of these parameters as the junctions were more distinct compared with those on the 7SEGA and FOURL test beams. Figures 2(c) to (e) illustrates the agreement found between MapCHECK 2, film, and the calculated dose for the 3ABUT test beam. Figures 2(c) and (e) highlight that, unlike the default model, the optimized model reproduces the peaks and troughs in film and MapCHECK 2‐measured dose at the junctions. Similar agreement between MapCHECK 2, film, and the optimized model were observed for the 7SEGA and FOURL test beams as indicated by the high gamma pass rates in Table 3. Dose at the junction of the 3ABUT test beam measured with ArcCHECK showed poor agreement with both the default and optimized models consistent with low gamma pass rates (Table 3). The ArcCHECK profiles displayed three dose ‘peaks’ in comparison to the optimized model (Fig. 2(c)). ArcCHECK appeared to overestimate the dose in the open field parts of the dose distributions compared with the calculated dose (Fig. 2(b)). The 7SEGA test beams showed a similar overresponse when measured with ArcCHECK.

### C. Leaf groove width adjustment using the FOURL test field

**Figure 3:**
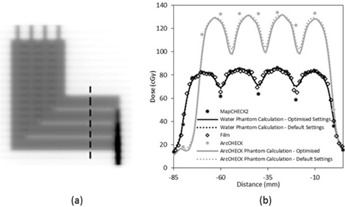
Dose distributions resulting from the FOURL test beam: (a) planar greyscale dose distribution; (b) dose profiles along the dashed lines in (a). Note: The water phantom calculation with the default and optimized settings overlay and make them indistinguishable.

The lower right section of the FOURL test beam dose distribution was used to analyze the model of the MLC groove (Fig. 3(a)). This section of the FOURL test beam produced three junctions formed by combining the field edges of alternate sides of the MLC. The dose at these junctions was influenced by the interleaf leakage parameter and the parameter that characterizes the width of the step in the MLC, referred to as the ‘leaf groove width’. At this stage in the MLC parameter optimization process, the calculated dose at these junctions was different to the calculated dose with the default MLC parameters as a result of previously increasing the interleaf leakage parameter from 5 to 25 (described in the Results section C). Changing the interleaf leakage caused the difference between calculated and both MapCHECK 2 and film‐measured dose to increase (junction dose increased). The leaf groove width was increased from 1.0 to 1.2 mm, which decreased the calculated junction dose and returned it to the same dose as calculated with the default MLC parameters. Despite the leaf groove width adjustment, no overall improvement in the match at the junctions was possible. Figure 3(b) shows the agreement between the MapCHECK 2, film, and the default and optimized models calculated dose. MapCHECK 2 measurements appeared to underestimate the dose compared with film by approximately 10%. ArcCHECK appeared to overestimate the dose and sampled the dips and peaks at irregular intervals, which made evaluation of the agreement difficult.

## IV. DISCUSSION

The ideal detector devices for adjusting MLC parameters should sample the detail of the test beam dose distributions sufficiently so feedback is provided as to which parameters should be adjusted. It was found that sampling the test beam dose distributions along lines of interleaf leakage and leaf transmission provided feedback for MLC parameter adjustment. MLCi MLCs project to leaf widths of 1 cm at isocenter and, since MapCHECK 2 possesses an orthogonal detector spacing of 1 cm, it was found that a single measurement taken with the MapCHECK 2 centered on the beam central axis did not sample both intraleaf transmission and interleaf leakage. The SNC patient software provides the ability to combine two sets of measurements and subsequently compare this with the TPS calculated dose distribution. The merged set of measurements (one centered and one offset by 0.5 cm) sampled the peaks and troughs of the test beam dose distributions which provided the information required to adjust the TPF MLC parameters. The distance the MapCHECK 2 is offset could be easily adapted for other MLC types.

ArcCHECK possesses a longitudinal detector spacing of 1.0 cm. The projected ArcCHECK detector spacing at isocenter varied when the ArcCHECK was centered on the isocenter as the detectors are located in a helical fashion. The variation in detector spacing at the isocenter results in irregular sampling of the test beams dose distributions peaks and troughs and made it difficult to understand how best to adjust the TPF MLC parameters. It is possible to adjust the SDD so as the peaks and troughs are sampled regularly, but this would vary for each test beam and so was deemed impractical.

ArcCHECK has been reported to overrespond to low energy scattered photons relative to an ion chamber^(19)^ giving rise to a field‐size‐dependent response. Field‐size correction factors are applied in an attempt to correct for ArcCHECK's overresponse.^15^, ^20^ However, even with field‐size correction factors applied, ArcCHECK appeared to overestimate the dose compared to the optimized model for the rectangular test fields whose long axis was parallel to the long axis of ArcCHECK (see 3ABUT dose profile in Fig. 2(b)). 3ABUT, 7SEGA, and DMLC test beams possessed rectangular segments and all showed an overresponse. This result is also consistent with the relatively high ArcCHECK gamma pass rates (Table 3) for the 10×10,20×20, and FOURL test beam which was less rectangular‐shaped. It is thought that, while the equivalent field‐size correction improves the response for square fields, the correction may breakdown for long rectangular fields.

The best match between MapCHECK 2‐measured and calculated dose in the MLC transmission region of the T‐shape field occurred when the leaf transmission was adjusted from 0.0120 to 0.007 and the interleaf leakage was adjusted from 5.00 to 25.00. Sikora^(6)^ reported leaf transmission and interleaf leakage values of 0.012 and 10.0 respectively for an MLCi MLC, but did not report details of how these values were determined. Lárraga‐Gutiérrez et al.^(21)^ showed that different detectors can give average MLC transmission differences of up to 12.5% (local difference). Due to the limited detail provided, it could not be confirmed if the difference between Sikora's proposed transmission values and the values determined in this study were a result of MLC manufacturing variations, the consequence of using a different detector or a combination of both. The leaf transmission and interleaf leakage parameters also affected the dose calculated for the FOURL and DMLC test beams. Agreement between MapCHECK 2, film, and the optimized model for these test beams all improved after adjustment of the leaf transmission and interleaf leakage parameters evident by the improved gamma pass rates shown in Table 3. ArcCHECK appeared to overrespond in the transmission region of the T‐shape field in comparison to calculated dose with the optimized model. This agrees with results reported by Chaswal et al.^(20)^ whom reported an overresponse of 10%–30% at distances of 1–8 cm from the field edge when compared to the TPS.

The dose tolerance on MLC transmission needs to be smaller for IMRT and VMAT compared with 3D CRT to achieve equivalent accuracy in dose to the target as the MLCs shadow the treatment area for a large proportion of the delivered MU. The Netherlands Commission on Radiation Dosimetry (NCRD) Report 22^(14)^ suggests dividing the tolerance used for the out‐of‐field region for 3D CRT by the modulation scaling factor (MSF) and apply this to IMRT. The NCRD recommends a tolerance of 10% between calculated and measured dose as a ‘state of the art value’. In the dose profiles examined (Fig. 1), the calculated dose of the optimized model was within 10% of the MapCHECK 2‐measured dose (local dose difference) for 33 out of the 36 points. Comparing the dose calculated with the default settings, only 17 out of the 36 points passed the 10% tolerance. In contrast, comparing ArcCHECK‐measured dose to the default and optimized model calculated dose, only 2 out of 17 and 1 out of 17 points passed the 10% tolerance, respectively.

The additional leakage at the junctions (interleaf) of the 3ABUT test beam dose distribution can be explained as leakage through the corner of the rounded MLCs.^(22)^ This additional dose was observed with both the film and MapCHECK 2 measurements. The ArcCHECK 3ABUT dose profile across the junction did not display the expected dose pattern. ArcCHECK showed a wave‐like pattern with a longer wavelength than that of the interleaf and intraleaf leakage pattern (Fig. 2(c)). The unexpected pattern was attributed to aliasing due to sampling the peaks and troughs at slightly greater than one full Period. The Period of the peaks and troughs in the dose distribution is 1 cm at isocenter due to the width of the MLCi MLCs. As the ArcCHECK was centered on the isocenter, the SDD, for the entrance detectors, was less than 100 cm resulting in projected detector spacing at isocenter greater than 1 cm. The three ‘peaks’ observed in Fig. 2(c) suggest that ArcCHECK overresponds to the dose in this region, which is consistent with the open parts of the field.

MapCHECK 2 underestimated the dose, relative to film, by approximately 10% at the junctions produced by the FOURL test beam. The junction dose is formed as a result of combining dose from step‐and‐shoot segments at locations where alternate sides of the MLCs fall. The junctions can be identified in Fig. 3(b) as three ‘troughs’ in the dose profile. The diodes in the ‘troughs’ are located in very steep dose gradients and the dose differences observed were attributed to volume averaging by the diode. It is therefore recommended that, for best results, a high resolution detector such as Gafchromic film (using a high dpi scan) should be used to evaluate FOURL test beam for the leaf groove width. All test beams were calculated with a fine dose calculation grid (0.15 cm) as higher values caused averaging at the sharp dose gradients, especially with the FOURL and interleaf leakage regions.

The TPF MLC adjustments improved the MapCHECK 2 gamma pass rates for all modulated test beams from the lowest pass rate being at 68.5% to at least 93.5%. The adjustments had relatively little effect on the clinical treatment fields (HIMRT and HDMLC) and indicated the default parameters provided a reasonable starting point. The changes in gamma pass rates for the ArcCHECK were variable for the modulated test beams and the pass rates ranged from 70.0%–97.9%. The gamma pass rates for both diode detector devices for the clinical test fields passed institutional tolerance (>95% at 3%/2 mm), but were lower for ArcCHECK than MapCHECK 2 (Table 3), which may be attributed to the overresponse to rectangular fields. These mismatches may have implications for PSQA of IMRT and VMAT treatment plans. For example, plans that possess characteristics that magnify the mismatches shown may result in false–positive QA results. Our institution currently uses MapCHECK 2 for PSQA measurement of IMRT plans and ArcCHECK for VMAT plans. Stemming from the results of this study, additional steps are taken in the event of VMAT plans failing PSQA measurements. If the plan is suspected of having a large number of rectangular segments, then MapCHECK 2 measurements are subsequently taken while attached to the gantry head. The MapCHECK 2 measurements are compared to calculation on a flat water phantom with gantry angles set to 0° for all control points. The comparison eliminates the ArcCHECK overresponse issue and helps to verify the accuracy of the MLC segment calculation and delivery.

The clinical impact of incorrectly modeled MLC transmission parameters depends on the proportion of dose to the volume of interest that results from transmission through the MLCs. The proportion of transmission is likely to vary from plan‐to‐plan for the target structures or organs examined. The variation in the contribution of transmission to dose throughout the patient makes it difficult to summarize the clinical impact of errors in transmission modeling. However, there are a number of studies which have presented results on the clinical impact of changes to MLC transmission.^2^, ^23^, ^24^, ^25^ Errors in MLC offset equate to systematically opening or closing the gaps between MLCs by a set distance. The impact of MLC gap‐width errors on a planned dose distribution depends on the plans average gap width, with small average gap‐width plans being more sensitive to MLC gap‐width errors.^(26)^ In a similar way to MLC transmission errors, it is difficult to summarize the clinical impact of errors in the MLC offset due to the variation in average gap width between plans. Nevertheless, the clinical significance of systematic MLC gap‐width errors have been reported for IMRT and VMAT for different clinical treatment sites.^26^, ^27^, ^28^, ^29^

It must be noted that there is an uncertainty associated with using the optimized model to evaluate the ArcCHECK performance as satisfactory matches achieved in a flat water phantom may not directly translate into accurate calculations in a cylindrical Perspex phantom. However, gamma pass rates for ArcCHECK versus calculation for the 10×10 and 20×20 test fields were greater than 98% for gamma criteria of 2% and 2 mm. Given the close agreement between the optimized model and ArcCHECK for simple open fields, it was considered that the magnitude of the differences observed in the ArcCHECK measurements for the modulated test fields were greater than the additional uncertainty associated with the cylindrical phantom.

## V. CONCLUSIONS

This study showed that the MapCHECK 2 was a suitable device for adjusting the TPF MLC parameters in Monaco XVMC, due to its accuracy and simple detector geometry. MapCHECK 2 measurements matched closely with film and the optimized MLC model, as indicated by high gamma pass rates for all MLC‐modulated test beams. Merging measurements that sampled the relevant parts of the dose distribution facilitated precise adjustment of the TPF MLC parameters. This technique maybe easily adapted for other MLC types. ArcCHECK was found to be less suitable for adjusting TPF MLC parameters due to its overresponse to rectangular fields and the resolution at which it sampled the test beam dose distributions, even using a ‘high density’ measurement. It is recommended that, where possible, MapCHECK 2 be used in preference to ArcCHECK to adjust the MLC parameters of the Monaco XVMC TPF MLC model.

## ACKNOWLEDGMENTS

I would like to acknowledge the support and funding of the St. Luke's Cancer Research Fund, the St. Luke's Radiation Oncology Network, and Dr. Luis Léon Vintro at University College Dublin School of Physics.

## COPYRIGHT

This work is licensed under a Creative Commons Attribution 3.0 Unported License.
